# Acupuncture for mild cognitive impairment: A systematic review with meta-analysis and trial sequential analysis

**DOI:** 10.3389/fneur.2022.1091125

**Published:** 2023-01-06

**Authors:** Zihan Yin, Yaqin Li, Cheng Jiang, Manze Xia, Zhenghong Chen, Xinyue Zhang, Ling Zhao, Fanrong Liang

**Affiliations:** ^1^School of Acu-Mox and Tuina, Chengdu University of Traditional Chinese Medicine, Chengdu, China; ^2^Acupuncture Clinical Research Center of Sichuan Province, Chengdu, China; ^3^Traditional Chinese Medicine Department, Deyang People's Hospital, Deyang, China

**Keywords:** acupuncture, mild cognitive impairment, systematic review, meta-analysis, trial sequential analysis (TSA)

## Abstract

**Background:**

There is insufficient evidence to support the use of acupuncture for mild cognitive impairment (MCI), and there is no consensus on its efficacy. This review aimed to determine the acupuncture effect in patients with MCI.

**Methods:**

Relevant and potentially eligible randomized controlled trials (RCTs) of acupuncture for MCI were obtained from four Chinese databases, four English databases, and additional resources up to 1 August 2022. The primary outcome was the improvement in overall cognitive function (OCF). Secondary outcomes were improved memory function (MF) and activities of daily living (ADLs). The revised Cochrane collaboration risk of bias (ROB) assessment tool (ROB 2.0) was applied to evaluate their methodological quality. The Review Manager software v 5.4 was used for analyses. Trial sequential analysis (TSA) 0.9.5.10 β software was used to estimate the required sample size and test the reliability of the pooled outcome. The quality of evidence was assessed using the Grading of Recommendations Assessment, Development, and Evaluation (GRADE) tool.

**Results:**

This meta-analysis included 11 RCTs with a total of 602 patients. The methodological quality of all trials was moderate. Low-quality evidence showed that acupuncture significantly improved OCF (Mini-Mental State Examination (MMSE): mean difference (MD) = 1.22, 95% confidence interval (CI): 0.78–1.66; the Montreal Cognitive Assessment Scale (MoCA): MD = 1.22, 95% CI: 0.47–1.97). In subgroup analyses, it was revealed that acupuncture significantly increased OCF in patients with MCI when compared to conventional medicine (CM) and sham acupuncture (SA). TSA's findings indicated that the evidence of improving OCF with acupuncture for patients with MCI was conclusive. Meanwhile, there is no statistical difference in the improvement of MF and ADL between acupuncture and CM. TSA showed that the evidence of improving MF and ADL for patients who had MCI and received acupuncture was inconclusive. The shreds of evidence of improving MF and ADL were ranked from low to critically low.

**Conclusion:**

Acupuncture appears to be an effective clinical application method for improving OCF in patients with MCI. However, due to low-quality evidence, more relevant and high-quality research is needed in this field.

**Systematic review registration:**

https://www.crd.york.ac.uk/prospero/display_record.php?ID=CRD42021291284, PROSPERO, No. CRD42021291284.

## Introduction

According to the World Health Organization report, more than 55 million people worldwide are living with dementia in 2021. By 2050, the total number of people affected by dementia is expected to reach 139 million (1, 2). Mild cognitive impairment (MCI) represents a possible prodrome of dementia (3, 4). The prevalence rate of in people over the age of 65 ranges from 3 to 42%, of which MCI in ~20% of them progress to develop dementia (5–7). Currently, the management of MCI has been extensively explored by researchers and clinicians, but treatment options are limited (8–10). Several studies illustrated that pharmaceutical treatments commonly applied to treat dementia might neither improve nor slow cognitive test performance decline in MCI patients (11, 12). Furthermore, these treatment options are expensive and pose health burdens associated with MCI control. Due to these factors, MCI is considered a prominent target symptom for early detection and intervention of dementia.

Acupuncture, part of traditional Chinese medicine, has been widely used as a non-pharmaceutical therapy for various cognitive dysfunctions, including MCI (13), postoperative cognitive impairment (14), vascular cognitive impairment (15), and so on. Numerous systematic reviews/meta-analyses (SRs/MAs) (13, 16–18) showed that acupuncture is an effective treatment for patients with MCI by improving cognitive function. Furthermore, several experimental studies demonstrated that acupuncture can enhance hippocampal synaptic transmission, inhibit neuroinflammation, release central neurotransmitters, and relieve oxidative stress (19–21).

There were numerous previous SRs**/**MAs (16–18); however, the sample size of the included randomized controlled trials (RCTs) from SRs**/**MAs was frequently small, and no research was conducted to estimate the sample size of the included studies, which might point out the risks of bias and false-positive results. Moreover, the quality of evidence derived from RCTs is unclear. These limitations obstructed the development of recommendations for clinical practice. Trial sequential analysis (TSA) might calculate the required information size (RIS) and assess futility boundaries to guide future trials (22). Therefore, in this study, MA and TSA of RCTs were conducted to evaluate acupuncture in the treatment of MCI to address the abovementioned issues, provide evidence for clinical applications, and serve as a reference for future clinical research.

## Methods

### Study design

This review protocol was registered in PROSPERO (No. CRD42021291284). SR/MA and TSA were implemented based on the Preferred Reporting Items for Systematic Review and Meta-Analysis (PRISMA) (23) and A Measure Tool to Assess Systematic Reviews-2 (AMSTAR-2) checklists (24).

### Inclusion and exclusion criteria

#### Types of studies

We included all peer-reviewed articles published on parallel RCTs and in English or Chinese. In contrast, quasi or cluster RCTs, non-RCTs, animal studies, letters, reviews, duplicated trails, and articles without data were excluded.

#### Types of participants

We included subjects with MCI who were diagnosed with specific criteria [such as Petersen's criteria (25), Jak/Bondi diagnosis (26), and National Institute on Aging-Alzheimer's Association (NIA-AA) criteria (27)]. Meanwhile, trials without detailed criteria were excluded.

#### Types of intervention group

The intervention group received acupuncture [using the insertion of needles into the specific acupoints (skin and underlying tissues)], as a monotherapy using manual/electronic/warm acupuncture, regardless of the acupoint selections.

#### Types of control group

The control group included conventional medicine (CM), which could enhance the cognitive ability of patients (such as Donepezil, Nimodipine, and Ginkgo biloba extract), regardless of the contents; and sham acupuncture (SA) (contact with skin without penetrating the exact acupoints; or insert into non-acupoints).

#### Types of outcome measures

The primary outcome measure was the improvement in cognitive function, as determined by the Montreal Cognitive Assessment Scale (MoCA) and the Mini-Mental State Examination (MMSE). The secondary outcome was the improvement in memory function (MF) and activities of daily living (ADLs). The inclusion criterion was any study that reported the primary or secondary outcome.

### Search strategy

Two reviewers independently performed a comprehensive search of the following electronic databases: four English databases [PubMed, Embase, Cochrane Central Register of Controlled Trials database (CENTRAL), and Web of Science (WOS)], four Chinese databases (China National Knowledge Infrastructure (CNKI), SinoMed Database (CBM), VIP Database, and WF Database), other included resources (Gray Literature Database (GLD), Allied and Complementary Medicine Database (AMED), WHO ICTRP, ClinicalTrials.gov, and ChiCTR), and published SRs on acupuncture for AD from inception to 1 August 2022. The following terms were imposed: (1) clinical conditions: MCI, cognitive dysfunction, etc.; (2) acupuncture-related words: acupuncture, acupoint, acupuncture plus moxibustion, electronic acupuncture, auricular acupuncture, etc.; and (3) trial type: a RCT. The terms “and” and “or” were combined between the search terms. Search strategies for these sources are shown in Appendix 1.

### Study selection and data extraction

All investigators received professional, evidence-based medicine training to implement this SR. After excluding duplicate articles and uploading potentially eligible studies to NoteExpress V.3.0, two reviewers (MX and ZC) independently screened the titles, abstracts, and keywords of all searched items. They identified the trials that met the abovementioned inclusion criteria. Divergences between the two reviewers were resolved through discussion between the two. A third party also assisted with the final decision.

Two reviewers independently accomplished data extraction using standardized tabulation. Extracted data included first author, publication date, country, standard of diagnosis, sample size, allocation ratio, age, gender, details of acupuncture and control groups, and outcomes. In case of any disagreement, the corresponding authors helped us in making a decision. Once the required information was inadequate, the corresponding author of the included studies was contacted.

### Quality assessment

Two investigators independently evaluated the risk of bias (ROB) using the revised Cochrane collaboration ROB assessment tool (ROB 2.0) (28). We evaluated the five items (randomization process bias, deviations from intended intervention bias, bias of missing outcome data, bias of measurement of an outcome, and the selection bias of the reported results). Each part was assessed and classified as high risk, low risk, or risk of some concern. The third intercessors were supposed to resolve any disagreements.

### Statistical analysis

For each study, we used pre- and post-differences or end-point scores as outcome indicators. The retrieved data were analyzed using the Review Manager (RevMan) version 5.4 software (Cochrane, London, UK). The Mantel–Haenszel method used the fixed-effects model for MAs, whereas the Der Simonian–Laired method used the random-effects model. The *I*^2^ statistic was used to identify and measure heterogeneity among RCTs. We considered heterogeneity when the *p*-value was < 0.1 and *I*^2^ was > 50%. In addition, all data were analyzed using a 95% confidence interval (CI). Mean differences (MDs) were applied for continuous data. To identify trials with possible heterogeneity, we used a subgroup analysis. Furthermore, when the number of included RCTs was > 2, potential reporting bias was explored using funnel plots.

### Trial sequential analysis

Conventional pairwise/cumulative MAs do not estimate the RIS using an empirical method. RIS was explored using TSA 0.9.5.10 β software (available at http://www.ctu.dk; Copenhagen Trial Unit, Denmark) to determine the sample size required for MA and explore whether the evidence included in our MA is credible and conclusive (29). *A priori* diversity-adjusted information size (α = 5%, *β* = 20%, relative risk reduction (RRR), and control event rates (CER) were calculated for each study) and an eventual breakthrough of the cumulative *Z*-curve of futility boundaries based on the O'Brien-Fleming alpha-spending function were used to estimate RIS with a critical value for a significant statistical effect of acupuncture.

### Quality of evidence

Subsequently, we used the Grading of Recommendations Assessment, Development, and Evaluation (GRADE) approach (30) to evaluate the quality of evidence and categorize it into four, namely, high, moderate, low, and critically low.

### Assessment of reviewer agreement

The intraclass correlation coefficient (ICC) was applied to measure the consistency between the two investigators using a κ value (κ score, 0.89) (31).

## Results

### Study description

#### Literature search

**Figure 1:**
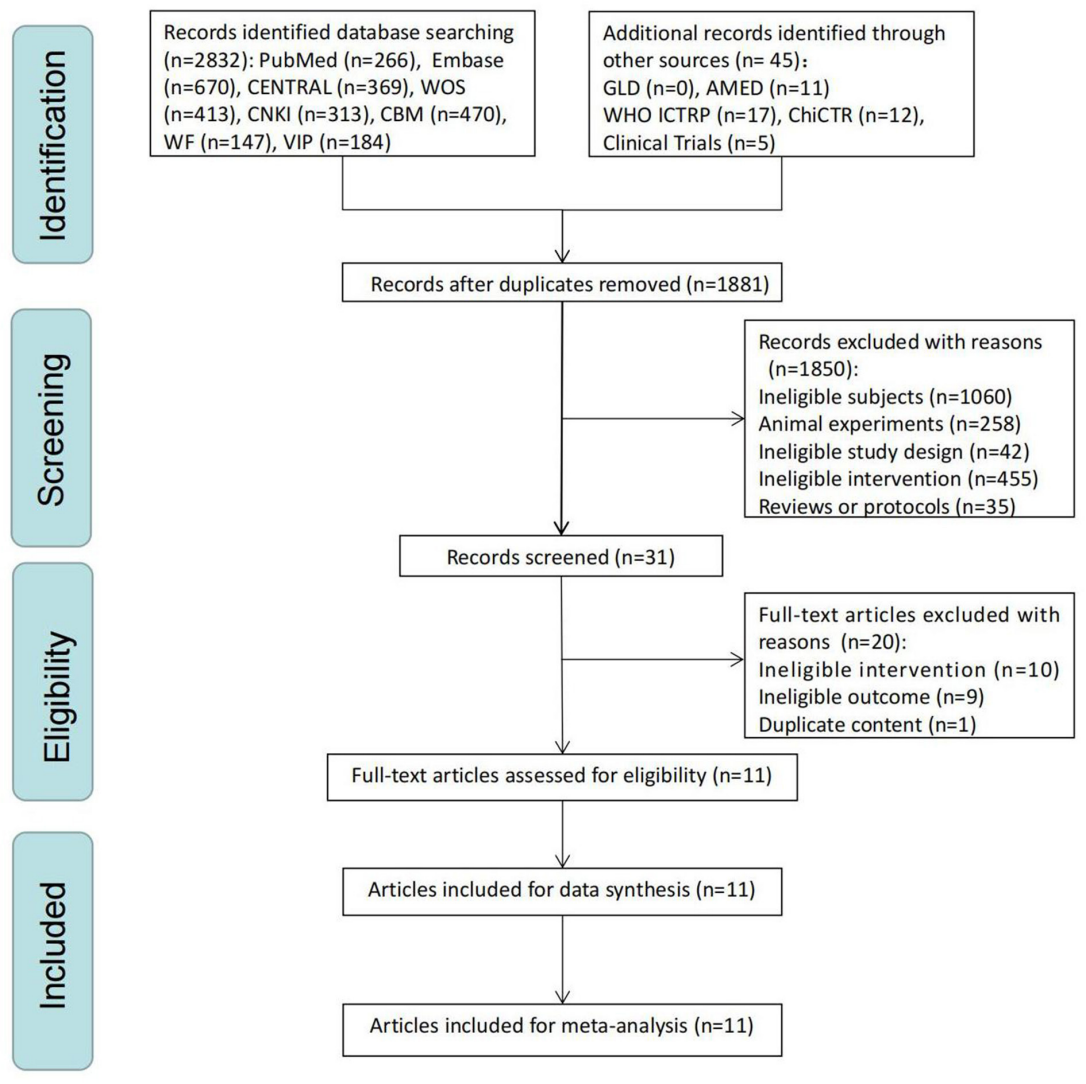
The Preferred Reporting Items for Systematic Review and Meta-Analysis (PRISMA) flow chart of a selection process.

Through comprehensive research, we identified 2,877 studies. After eliminating duplicate trials, we had 1,881 studies. After the initial screening, 31 articles remained. Finally, after reading the full-text articles, 19 papers were excluded (10 studies with non-acupuncture, nine with ineligible outcomes, and one with duplicate content), leaving 11 RCTs (32–42). The flowchart of PRISMA is depicted in Figure 1, and the reasons for the exclusion of full-text studies are detailed in Appendix 2.

#### Study characteristics

Table 1 presents the basic characteristics of 11 studies included in MA. A total of 11 trials with 602 subjects were implemented in China. Only one study (39) had a sample size of more than 100, whereas the other studies had low sample sizes. In the studies, 1:1 was regarded as a generally applied allocation ratio. The Diagnostic and Statistical Manual of Mental Disorders (DSM) or Petersen's criteria were frequently used for diagnosis. In addition, the mean patient age is 60–80 years, and the male-to-female ratio is largely consistent. Meanwhile, CM was the most commonly used comparator intervention. The course of the disease was reported in nine articles. The most documented outcome was an improvement in the MMSE score. A follow-up period was not covered in any of the articles.

**Table 1:** Main characteristics of included randomized controlled trials (RCTs).

**Study**	**Country**	**Sample size**	**Allocation ratio**	**Diagnostic criteria**	**Age**	**Gender (M/F)**	**Course of the disease (month)**	**(A)**	**(B)**	**Follow-up period**	**Efficacy criteria**	**Main Results**
								**Treatment Group**	**Control Group**			
Zheng et al. (32)	China	60	1:1	①	A: 63.52 ± 6.20 B: 66.00 ± 6.42	A: 9/16 B: 7/17	A: 38.52 ± 22.2 B: 42.84 ± 15.48	Acupuncture	CM (6 ml/day dose of Ginkgo biloba extract drops)	/	1. MMSE 2. MoCA	1. A>B 2. A>B
Tan et al. (34)	China	32	1:1	②	A: 65.88 ± 4.66 B: 64.56 ± 5.25	A: 10/6 B: 6/10	/	Acupuncture	Sham acupuncture	/	1. MMSE 2. MoCA	1. A>B 2. A>B
Xu and Peng (33)	China	60	1:1	③	A: 62.12 ± 8.01 B: 61.20 ± 7.63	A: 16/14 B: 16/14	A: 8.55 ± 3.10 B: 9.60 ± 2.72	Acupuncture	CM (60 mg/day dose of Nimodipine)	/	1. MMSE 2. MoCA	1. A>B 2. A>B
Liu (35)	China	32	15:17	④	A: 73.00 ±7.67 B: 77.22 ±5.65	A: 9/6 B: 9/8	/	Acupuncture	CM (2.5 mg/day dose of Donepezil)	/	1. MMSE 2. CMS	1. A>B 2. A=B
Du (36)	China	40	1:1	④	A: 69.03 ± 5.47 B: 68.25 ± 5.80	A: 11/9 B: 13/7	A: 10.08 ± 2.79 B: 10.01 ± 2.39	Acupuncture	CM (5 mg/day dose of Donepezil)	/	1. MMSE 2. MoCA	1. A>B 2. A>B
Zhu et al. (37)	China	60	1:1	⑤	A: 62.13 ± 7.99 B: 60.90 ±7.52	A: 17/13 B: 16/14	A: 8.73 ± 3.05 B: 9.50 ± 2.82	Acupuncture	CM (90 mg/day dose of Nimodipine)	/	1. MMSE 2. MoCA	1. A>B 2. A=B
Zhu et al. (38)	China	60	1:1	⑥	A: 45-75 B: 46-74	A: 19/11 B: 21/9	A: 3-17 B: 6-16	Acupuncture	CM (90 mg/day dose of Nimodipine)	/	1. MMSE 2. MoCA 3. ADL	1. A>B 2. A>B 3. A>B
Zhao et al. (39)	China	192	1:1	①	A: 69 ± 7 B: 67 ± 6	A: 46/48 B: 44/49	/	Acupuncture	CM (90 mg/day dose of Nimodipine)	/	MMSE	A>B
Liu (40)	China	36	17:19	④	A: 66.00 ± 6.84 B: 69.32 ± 6.86	A: 7/10 B: 9/10	A: 27.84 ± 7.94 B: 30 ± 11.11	Acupuncture	CM (2.5 mg/day dose of Donepezil)	/	MMSE	A>B
Jin (41)	China	30	7: 8	②	A: 72.54 ± 7.07 B: 73.67 ± 3.27	A: 8/6 B: 9/7	A: 30.24 ± 10.25 B: 31.10 ± 10.39	Acupuncture	CM (90 mg/day dose of Nimodipine)	/	1. MoCA 2. CMS	1. A>B 2. A=B
Wang (42)	China	60	1:1	②	A: 65.80 ± 5.08 B: 65.57 ± 5.22	A: 16/14 B: 17/13	A: 7.80 ± 2.52 B: 7.83 ± 2.46	Acupuncture	CM (90 mg/day dose of Nimodipine)	/	1. MMSE 2. ADL	1. A=B 2. A=B

① International Working Group criteria; ② Petersen RC. Mild cognitive impairment: Clinical characterization and outcome. Arch Neurol 1999, 56, 303-308; ③ National Institute of Neurological and Communicative Disorders and Stroke and the Alzheimer's Disease and Related Disorders Association criteria; ④ Diagnostic and Statistical Manual of Mental Disorders (DSM); ⑤ Guidelines for the diagnosis and treatment of dementia and cognitive Impairment in China; ⑥ Expert consensus on the prevention and treatment of Cognitive Impairment in China; CM, conventional medicine; MMSE, Mini-Mental State Examination; MoCA, Montreal Cognitive Assessment Scale; CMS, Clinical Memory Scale; ADL, activity of daily living.

#### Acupuncture details

According to the Standards for Reporting Interventions in Clinical Trials of Acupuncture (STRICTA) (43), acupuncture details are summarized in Table 2. In the acupuncture rationale, all the included 11 RCTs were mentioned. In the section on acupuncture details, the number of needle insertions per participant in each session of included studies ranged from 3 to 18; Baihui (GV 20) and Sishencong (EX-HN1) were the frequently applied acupuncture points; acupuncture insertion depth was 7.5–40 mm; Deqi was mentioned in 10 studies; the electronic method was applied to five studies, the manual method to four articles, and the warm method to two articles. The most frequently used brand of acupuncture was Hwato, and the diameter and length of a needle were 0.30 and 25/40 mm, respectively. The electroacupuncture apparatus was G6805. For treatment regimens, the number of treatment sessions ranged from 20 to 60; the frequency of treatment sessions was daily, and the session duration of acupuncture ranged from 28 to 180 days. In other components, three researchers documented the details of other interventions; only one article provided a detailed setting and treatment context. Notably, only one study (34) described details about acupuncturists. In the section on comparator interventions, eight trials documented the rationale for a comparator; all trials had a complete description of the control/comparator.

**Table 2:** Details of acupuncture methods according to Standards for Reporting Interventions in Clinical Trials of Acupuncture (STRICTA).

**Study**	**Acupuncture rationale**			**Details of needling**							**Treatment regimen**		**Other components**		**Practi-tioner**	**Comparator interventions**	
	**1a**	**1b**	**1c**	**2a**	**2b**	**2c**	**2d**	**2e**	**2f**	**2g**	**3a**	**3b**	**4a**	**4b**	**5**	**6a**	**6b**
Zheng et al. (32)	TCM	Y	NA	10	GV 24, GB 20 (bilateral), GV 23, GV 29, LI 20 (bilateral), LI 4 (bilateral), ST 36 (bilateral)	15-40 mm	Deqi	Manual	40 min	Diameter and length: 0.30 mm & 40 mmNeedle brand: Zhongyan-Electroacupuncture apparatus: 6805C	60	Frequency: five times per week Duration: 84 days	NA	NR	NR	Y	Y
Tan et al. (34)	TCM	Y	NA	13	EX-HN1, GV 29, PC 6 (bilateral), KI 3 (bilateral), ST 40 (bilateral), and LR 3 (bilateral)	15 mm	NR	Manual	NR	Diameter and length: 0.35 mm & 25 mmNeedle brand: Hwato	20	Frequency: five times per week Duration: 12 weeks	NA	NR	Y	Y	Y
Xu et al. (33)	TCM	Y	NA	8	GV 20, EX-HN 1, GV 24, GB 20 (bilateral)	12.5-30 mm	Deqi	Electronic	NR	Diameter and length: 0.30 mm & 40 mmNeedle brand: Zhongyan-Electroacupuncture apparatus: 6805C	24	Frequency: three times per week Duration: 56 days	NA	NR	NR	Y	Y
Liu (35)	TCM	Y	NA	14	EX-HN 1, GB 20 (bilateral), GB 39 (bilateral), BL 23 (bilateral), KI3 (bilateral), HT 7 (bilateral)	7.5-25mm	Deqi	Electronic	20 min	Diameter and length: NRNeedle brand: NR-Electroacupuncture apparatus: G6805-II	30	Frequency: each day Duration: 30 days	NA	NR	NR	NR	Y
Du (36)	TCM	Y	NA	18	GV 24, GV 14, GV 23, GV 29, GV 16, GV 15, EX-HN 1, KI3 (bilateral), KI4 (bilateral), ST 36 (bilateral), GB 39 (bilateral)	15-40 mm	Deqi	Manual	40 min	Diameter and length: 0.30 mm & 25/40 mmNeedle brand: Tianxie-Electroacupuncture apparatus: 6805C	48	Frequency: six times per week Duration: 56 days	Y	NR	NR	Y	Y
Zhu et al. (37)	TCM	Y	NA	4	GV 24, GV 14, GB 39 (bilateral)	≤ 50 mm	Deqi	Warm	30 min	Diameter and length: 0.3 mm & 25/40 mmNeedle brand: Hwato	56	Frequency: six times per week Duration: 56 days	Y	NR	NR	NR	Y
Zhu et al. (38)	TCM	Y	NA	3	GV 24, CV 4, GV 4	25 mm	Deqi	Warm	30 min	Diameter and length: 0.3 mm & 50 mmNeedle brand: Hwato	60	Frequency: six times per week Duration: 70 days	NA	NR	NR	NR	Y
Zhao et al. (39)	TCM	Y	NA	8	GV 20, EX-HN 1, GV 24, GB 20 (bilateral)	15-25 mm	Deqi	Electronic	30 min	Diameter and length: 0.3 mm & 25/40 mmNeedle brand: Hwato-Electroacupuncture apparatus: G6805-II	24	Frequency: three times per week Duration: 56 days	Y	Y	NR	Y	Y
Liu (40)	TCM	Y	NA	9	GV 20, GB 20 (bilateral), BL 23(bilateral), GB 39 (bilateral), KI3 (bilateral)	13-25 mm	Deqi	Electronic	NR	Diameter and length: 0.35 mm & 25 mmNeedle brand: Shenlong-Electroacupuncture apparatus: G6805-II	30	Frequency: each day Duration: 30 days	NA	NR	NR	Y	Y
Jin (41)	TCM	Y	NA	10	EX-HN 1, GB 20 (bilateral), BL 23(bilateral), KI3 (bilateral)	13-25 mm	Deqi	Electronic	20 min	Diameter and length: 0.30 mm & 40 mmNeedle brand: Hwato-Electroacupuncture apparatus: G6805-II	45	Frequency: each day Duration: 45 days	NA	NR	NR	Y	Y
Wang (42)	TCM	Y	NA	12	GV 20, EX-HN 1, GV 24, GB 20 (bilateral), BL 23(bilateral), KI3 (bilateral)	25 mm	Deqi	Manual	30 min	Diameter and length: 0.38 mm & 25-65 mmNeedle brand: Hwato	28	Frequency: each day Duration: 28 days	NA	NR	NR	Y	Y

1a, Style of acupuncture; 1b, Reasoning for the treatment provided; 1c, Extent to which treatment was varied; 2a, Number of needle insertions per subject per session; 2b, Names of points used; 2c, Depth of insertion; 2d, Response sought; 2e, Needle stimulation; 2f, Needle retention time; 2g, Needle type; 3a, Number of treatment sessions; 3b, Frequency and duration of treatment sessions; 4a, Details of other interventions administered to the acupuncture group; 4b, Setting and context of treatment; 5, Description of participating acupuncturists; 6a, Rationale for the control or comparator; 6b, A precise description of the control or comparator; TCM, traditional Chinese medicine; NA, not applied; NR, not recorded; Y, Yes; &, and.

### Quality assessment

**Figure 2:**
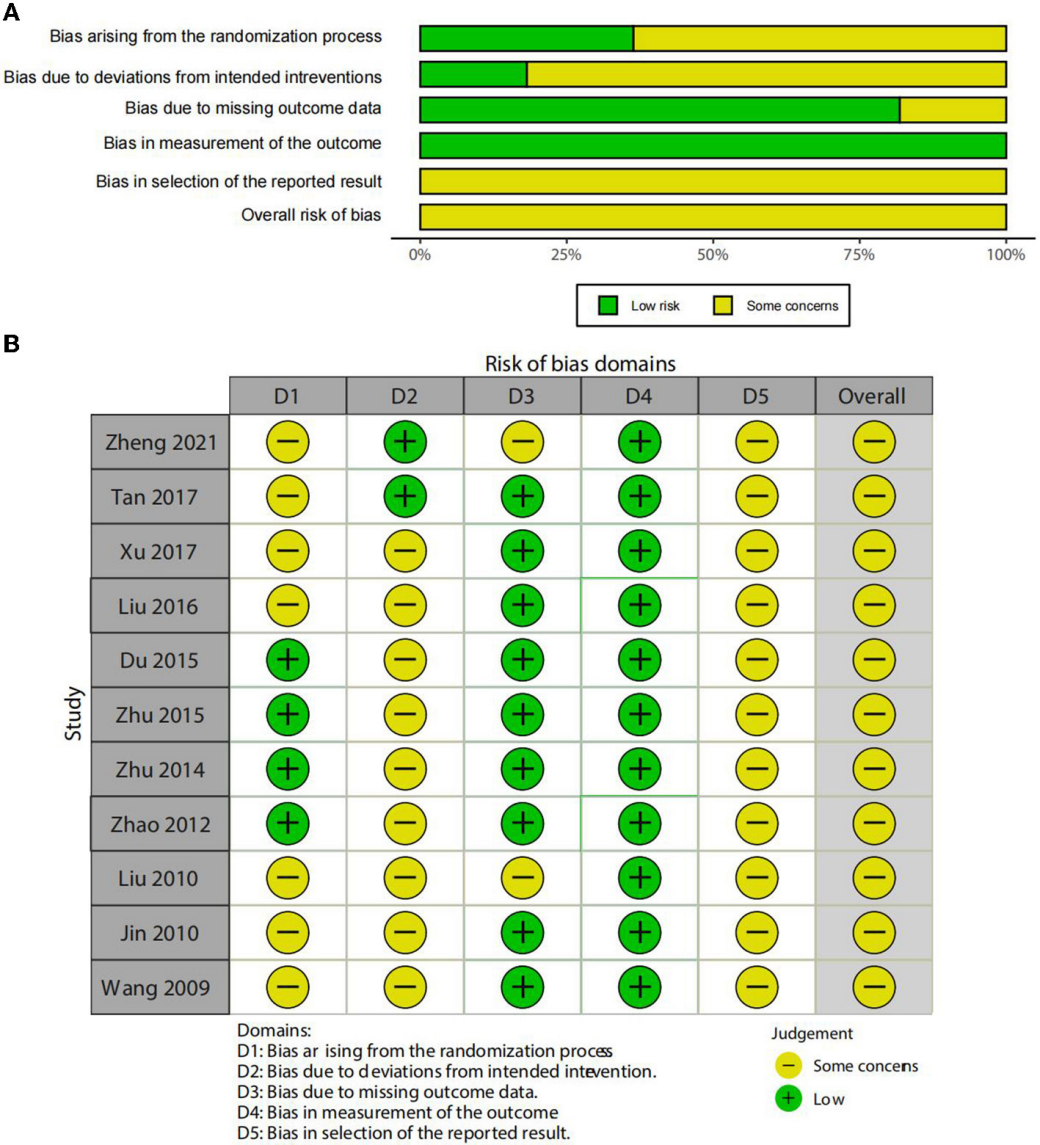
**(A)** Risk of bias graph and **(B)** Risk of bias summary.

The risk of bias of RCTs was assessed using RoB 2. Meanwhile, ROB graphs were performed using the Shiny app (https://mcguinlu.shinyapps.io/robvis/). In the section on the randomization process, although all articles documented that they were randomized, only four trials (36–39) were ranked as low risk and the rest of the trials had certain concerns due to the generation of an unclear random sequence. For deviations from the intended interventions, only two studies (32, 34) were ranked as low risk, the remaining studies were ranked as the risk of some concerns due to their short descriptions. For missing outcome data, two trials (32, 40) had a risk of some concerns. All trials were ranked as low risk in the measurement of an outcome. Notably, in the selection of the reported results, all RCTs were assessed for some concerns due to a lack of a protocol/registration about a pre-specified analysis plan. Figure 2 illustrates the ROB results.

### Effects of intervention

#### Primary outcome: Improvement in overall cognitive function

##### Improvement in MMSE

**Figure 3:**
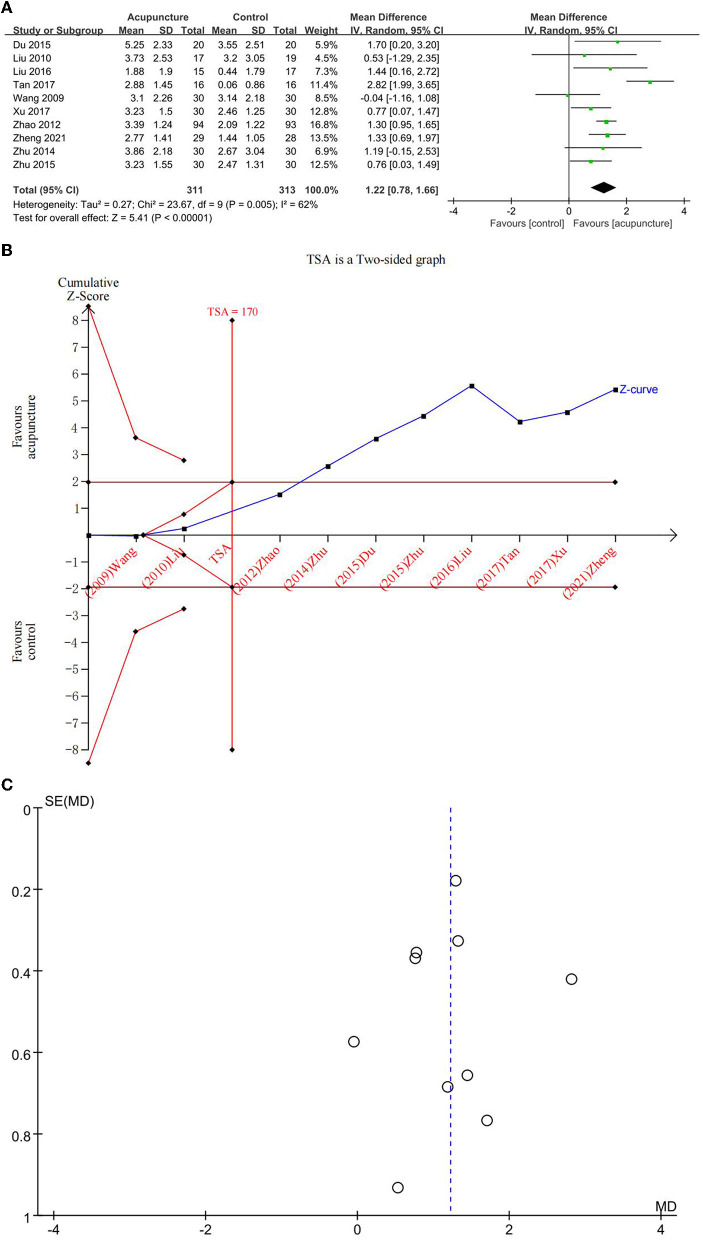
**(A)** Forest plot of improvement of Mini-Mental State Examination (MMSE); **(B)** trial sequential analysis of improvement of MMSE; and **(C)** a funnel plot of improvement of MMSE.

We explored 10 RCTs for the beneficial effects of acupuncture on overall cognitive function (OCF) in subjects with MCI using MMSE (Figure 3A). Differences between acupuncture and comparator interventions were statistically significant (MD = 1.22; 95% CI: 0.78, 1.66). Due to the existence of heterogeneity (*I*^2^ = 62%, *p* = 0.005), a random-effects model was applied. In a comparison between acupuncture and the comparator intervention, TSA revealed that the cumulative *Z*-curve had crossed the RIS boundary (RIS = 170), indicating that the sample size was sufficient to determine whether acupuncture treatment was superior to comparator interventions in improving MMSE on MCI (Figure 3B). As illustrated in Figure 3C, a funnel plot manifested a low ROB of publication. The GRADE approach evaluated that the quality of evidence in improving MMSE was “low” (Table 3).

**Table 3:** Quality of evidence included RCTs by Grading of Recommendations Assessment, Development, and Evaluation (GRADE).

**Outcomes**	**Included RCTs (patients)**	**Relative effect (95% CI)**	**Quality assessment**								**Quality of evidence**
			**Risk of bias**	**Inconsistency**	**Indirectness**	**Imprecision**	**Publication bias**	**Large effect**	**Dose response**	**All plausible confounding**	
MMSE	10 (592)	MD 1.22 (0.78, 1.66)	Serious①	Serious②	Not serious	Not serious	Undetected	Undetected	Undetected	Undetected	Low
MoCA	7 (339)	MD 1.22 (0.47, 1.97)	Serious①	Serious②	Not serious	Not serious	Undetected	Undetected	Undetected	Undetected	Low
CMS	2 (62)	MD 6.05 (-1.33, 13.44)	Serious①	Not serious	Not serious	Serious③	Undetected	Undetected	Undetected	Undetected	Low
ADL	2 (120)	MD 3.19 (-4.53, 10.90)	Serious①	Serious②	Not serious	Serious③	Undetected	Undetected	Undetected	Undetected	Critically Low

① Most information is from the moderate risk studies, and there are major limitations; ② The size and direction of the effect size, the overlap of the confidence interval (CI) is small, the P-value of the heterogeneity test is small, and the combined results of I^2^ value are large; ③ The sample is insufficient.

Due to significant heterogeneity, a subgroup analysis was applied. Given that control groups (CM and SA) were different, a subgroup analysis was performed. Heterogeneity was significantly reduced (*I*^2^ = 7%, *p* = 0.37), and a fixed-effects model was applied. The results of the MA found statistical differences between acupuncture and CM (MD = 1.09, 95% CI: 0.83, 1.36). The MA results found statistical differences between acupuncture and SA (MD = 2.82, 95% CI: 1.99, 3.65). These findings demonstrated that acupuncture increased MMSE scores more effectively than CM and SA.

##### Reduction of MoCA

**Figure 4:**
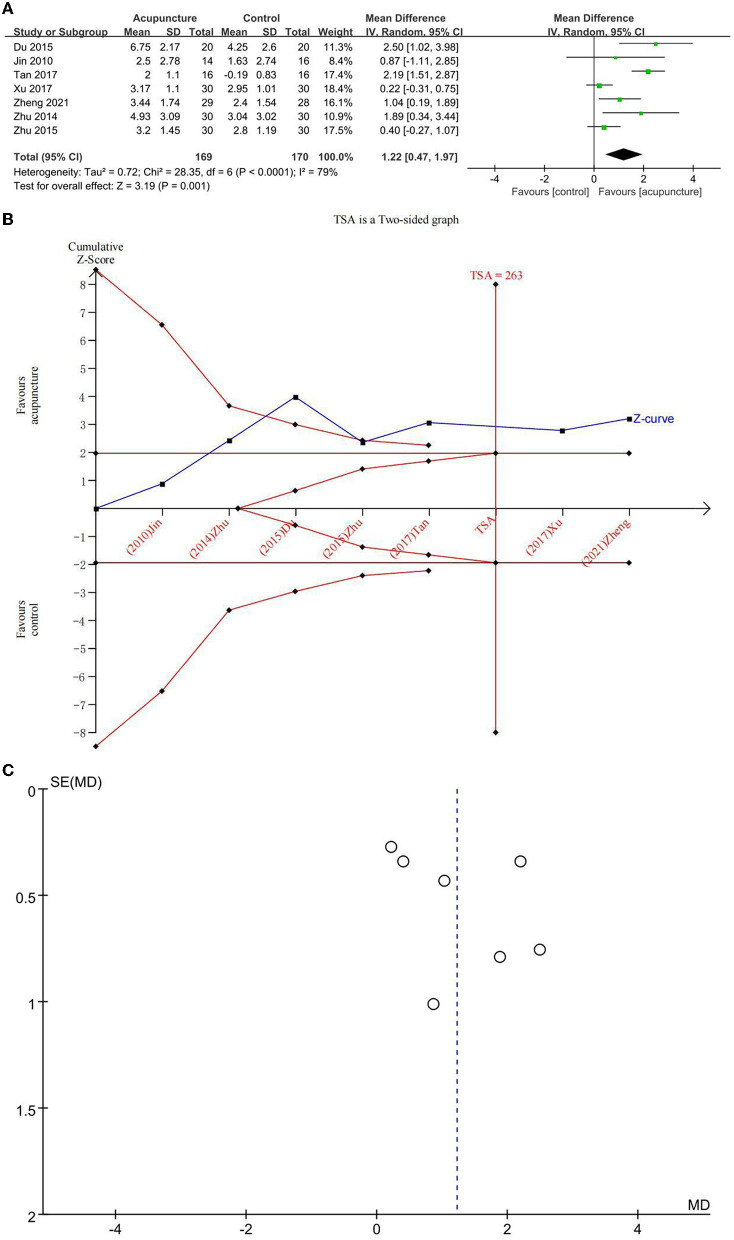
**(A)** Forest plot of improvement of Montreal Cognitive Assessment Scale (MoCA); **(B)** trial sequential analysis of improvement of MoCA; and **(C)** funnel plot of improvement of MoCA.

Seven studies explored the beneficial effects of acupuncture on OCF in subjects with MCI using MoCA (Figure 4A). The difference between acupuncture and comparator interventions was statistically significant (MD = 1.22; 95% CI: 0.47, 1.97). Due to major heterogeneity (*I*^2^ = 79%, *p* = 0.001), a random-effects model was used. In a comparison between acupuncture and the comparator intervention, TSA revealed that the cumulative *Z*-curve had crossed an RIS boundary (RIS = 263), indicating that the sample size was competent to determine whether acupuncture treatment was superior to comparator interventions in improving MoCA on MCI (Figure 4B). As displayed in Figure 4C, the funnel plot manifested a low ROB of publication. The GRADE approach evaluated that the quality of evidence regarding MoCA improvement was “low” (Table 3).

Because control groups (CM and SA) were different, a subgroup analysis was implemented. The heterogeneity was significantly reduced (*I*^2^ = 59%, *p* = 0.004). The results of MA found statistical differences between acupuncture and CM (MD = 0.93, 95% CI: 0.29, 1.56) as well as between acupuncture and SA (MD = 2.19, 95% CI: 1.51, 2.87). These findings demonstrated that acupuncture increased MoCA scores better than CM and SA.

#### Secondary outcome: Improvement in MF

**Figure 5:**
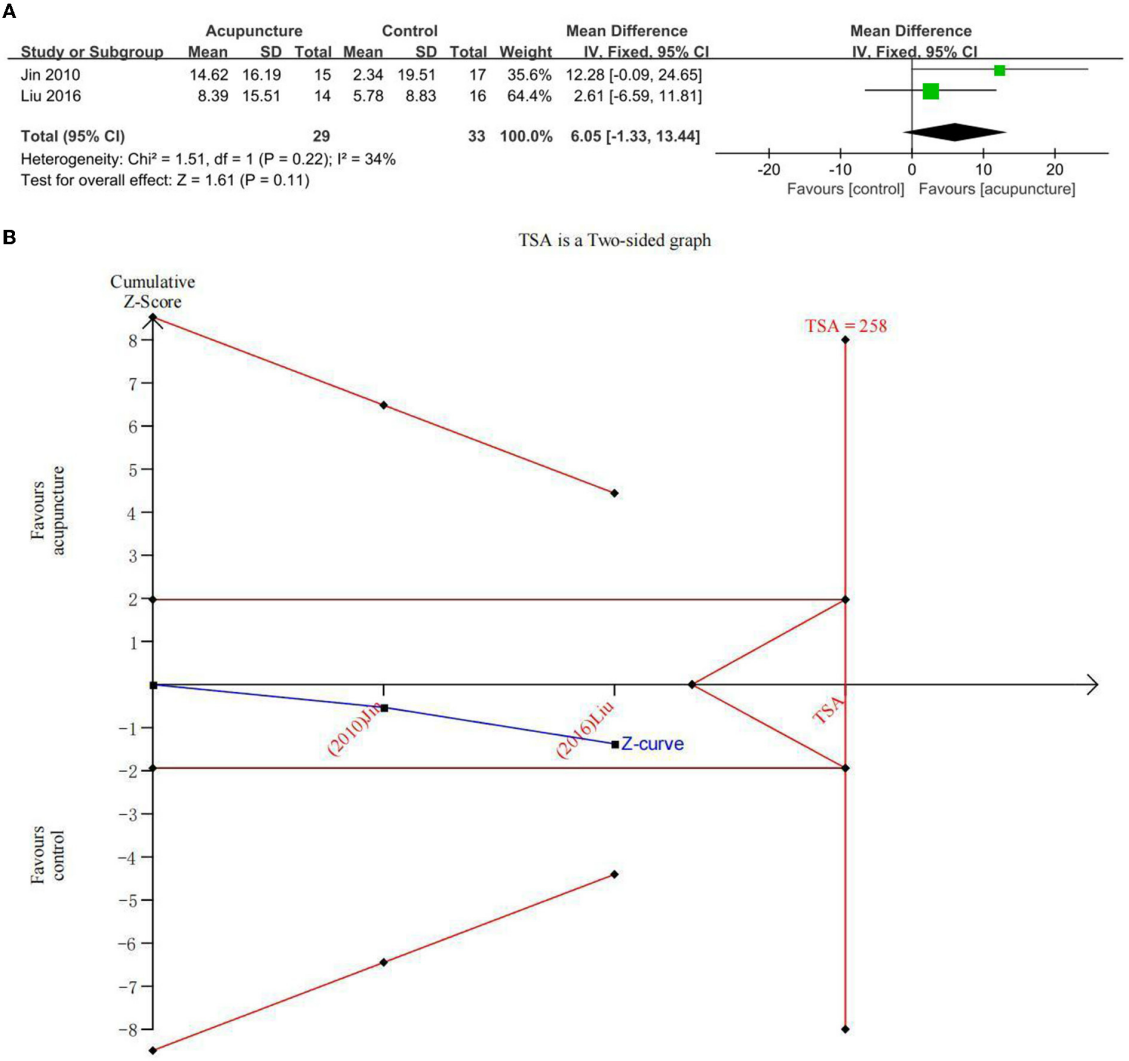
**(A)** Forest plot of improvement of clinical memory scale (CMS) and **(B)** trial sequential analysis of improvement of CMS.

Two studies explored the beneficial effects of acupuncture on MF in subjects with MCI using a clinical memory scale (CMS) (Figure 5A). There was no statistical difference between acupuncture and the comparator intervention (CM) (MD = 6.05; 95% CI: −1.33, 13.44). Due to low heterogeneity (*I*^2^ = 34%, *p* = 0.22), a fixed-effects model was used. In the comparison between acupuncture and CM, TSA investigated that the cumulative *Z*-curve had not crossed the RIS boundary (RIS = 258), indicating that the sample size was insufficient to determine whether acupuncture treatment was equal to comparator interventions in improving CMS on MCI (Figure 5B). The GRADE approach evaluated that the quality of evidence regarding CMS improvement was “low” (Table 3).

#### Secondary outcome: Improvement in ADL

**Figure 6:**
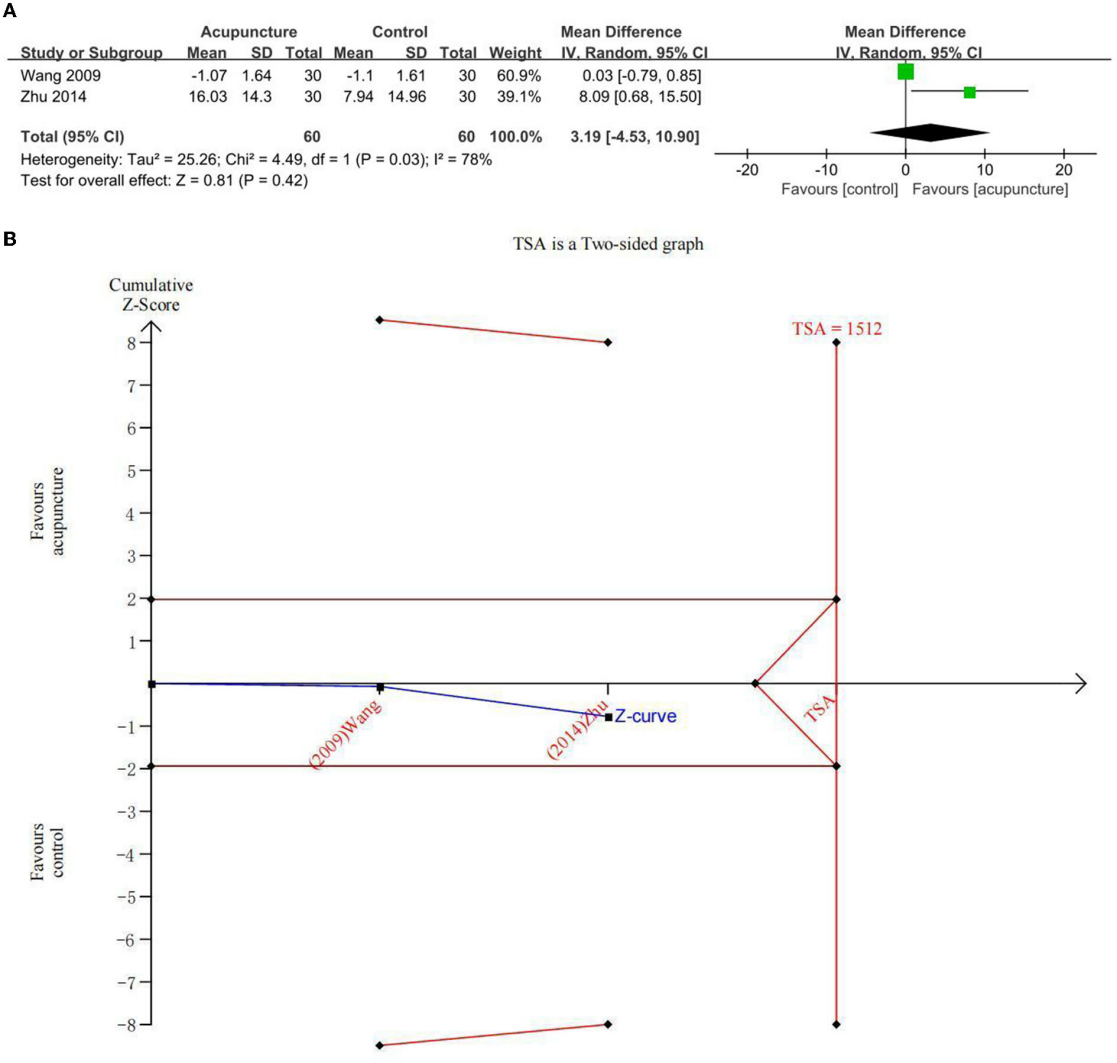
**(A)** Forest plot of improvement of activity of daily living (ADL) and **(B)** trial sequential analysis of improvement of ADL.

Two studies explored the beneficial effects of acupuncture on ADL in subjects with MCI using the ADL scale (Figure 6A). There was no statistical difference between acupuncture and the comparator intervention (CM) (MD = 3.19; 95% CI: −4.53, 10.90). Due to major heterogeneity (*I*^2^ = 78%, *p* = 0.03), a random-effects model was used. In the comparison between acupuncture and comparator intervention, TSA investigated that the cumulative *Z*-curve had not crossed the RIS boundary (RIS = 1,512), indicating that the sample size was insufficient to determine whether acupuncture treatment was equal to the comparator intervention in improving ADL on MCI (Figure 6B). The GRADE approach evaluated that the quality of evidence regarding ADL improvement was “critically low” (Table 3).

### Quality of evidence

The GRADE approach was employed to measure the quality of evidence from 11 RCTs (Table 3). The analysis assessed the four outcome measurements: MMSE improvement, MoCA improvement, CMS improvement, and ADL improvement. Overall, pieces of evidence were ranked from low to critically low in quality. The results indicated that none of the outcomes had high-quality evidence, two (1/2, 50%) had low-quality evidence, and the other two (1/2, 50%) had critically low-quality evidence. The ROB and inconsistency were the main reasons for low-quality evidence.

## Discussion

Mild cognitive impairment, a vital stage between normal aging and dementia, is one of the key health issues for sufferers, families, and society of an aging population worldwide. In recent years, acupuncture has been widely applied for the treatment of MCI. However, previous studies (13, 16, 17, 44) have reported that more carefully designed studies are needed to confirm the efficacy of acupuncture for MCI; its effectiveness is uncertain because it is unknown whether the sample size is sufficient and the quality of the evidence is high. This review includes 11 studies with 602 participants to summarize and analyze the findings of acupuncture for MCI.

### Summary of main findings

All RCTs had moderate methodological quality, as determined by the ROB 2.0 approach. These articles compared acupuncture with controls (including CM and SA). This MA included four outcomes (improvement in MMSE, MoCA, CMS, and ADL) and illustrated the following results. For instance, for improving OCF, acupuncture demonstrated statistically significant differences in improving MMSE and MoCA scores; in subgroup analyses, acupuncture significantly improved in comparison with CM and SA. Meanwhile, TSA analysis demonstrated sufficient information size; however, pieces of evidence were ranked as low. Regarding CMS and ADL, we found no differences between acupuncture and CM in MA; TSA showed that the amount of information was insufficient and the quality of evidence was ranked as critically low.

### Strengths and limitations

To our knowledge, this study is the first MA with TSA to certify acupuncture therapy for improving OCF, MF, and the activities of daily living with MCI. Meanwhile, we used ICC to measure the consistency of investigators. TSA was used to provide the required sample size and a conclusive result by maximizing the use of extracted data in the included trials. The GRADE approach was applied to evaluate the quality of evidence from the included RCTs. As a result, our findings might provide credible evidence, which may guide clinical treatment decisions and health policies on MCI. Nonetheless, the following shortcomings should be considered. A significant limitation of this study is the low quality of evidence of the included RCTs. Meanwhile, the characteristics of the subjects and acupuncture methods of the included studies were insufficiently homogeneous. Furthermore, all included studies were carried out in China, which might potentially contribute to bias. Additionally, acupuncture has a long-term effect (45–49); however, no studies had a follow-up period, which is needed to illuminate the long-term effect of acupuncture on MCI.

### Implications for clinical practice

Diagnostic and Statistical Manual of Mental Disorders (DSM) and Petersen's criteria are clinically and frequently applied for the diagnosis of MCI. Nevertheless, the Jak/Bondi diagnosis criteria are suggested to be better than the two due to their individual phenotype and accurate diagnosis. In detail, acupuncture utilizes a variety of acupoints. However, the most frequently applied acupoints are GV 20 and EX-HN1, the response sought is Deqi, the commonly used needle stimulation is electroacupuncture, the needle type used is Hwato (0.35 and 25/40 mm), and the main frequency is daily. Despite the flaws of MA, all included RCTs demonstrated that acupuncture improved OCF in managing MCI. According to therapeutic effects, it should be considered in clinical practice when clinical decision-makers consider a selection. In addition, based on existing findings, acupuncture therapy is not inferior to CM in improving MF and ADL, thus, for patients who have MCI and are either unresponsive to CM or poly-pharmacy, acupuncture therapy should be administered.

### Implications for future research

The findings of this study have a number of important implications for further research. First, the methodological quality of the included trials is moderate due to the problems with the randomization process, deviations from the intended interventions, and the selection of the reported results. Therefore, the randomized design, blinded method, and others of future RCTs may conform discreetly to the Cochrane ROB 2.0 tool. Meanwhile, this study collected and summarized acupuncture details according to STRICTA, and we found that most of the included RCTs neglected the details of other components, practitioner, and comparator interventions. As a result, STRICTA and Consolidated Standards of Reporting Trials (CONSORT) (50) should be considered as the standard criteria for future RCTs using acupuncture therapy. Moreover, TSA analysis indicated that the sample size was adequate in terms of acupuncture improving OCF; nevertheless, due to the limited quality of evidence, future high-quality research on this aspect should be implemented. Meanwhile, in this study, acupuncture can play a pivotal role in improving global cognitive function. To the best of our knowledge, the global cognitive function has been divided into memory, verbal, executive functions and so on. However, evidence for the efficacy of acupuncture on these specific cognitive functions is very scarce. Thus, the efficacy of acupuncture for certain MCI symptoms needs to be confirmed in the future. In addition, evidence for the improvement of ADL with acupuncture in patients with MCI needs to be explored in the future. Additionally, this study shows the short-term efficacy of acupuncture; however, the long-term efficacy of acupuncture for MCI remains unclear due to the paucity of follow-up studies. Therefore, future studies should expand the sample size, increase the study period, and extend a follow-up period to assess long-term sustained efficacy. Finally, the potential mechanisms of acupuncture for MCI should be studied further.

## Conclusion

According to the existing research, the use of acupuncture in the treatment of patients with MCI demonstrated promising efficacy. However, due to low-quality evidence, further studies are still required to confirm the findings in the following years.

## Data availability statement

The original contributions presented in the study are included in the article/Supplementary material, further inquiries can be directed to the corresponding author/s.

## Author contributions

Conceptualization: ZY, LZ, and FL. Methodology, software, and writing—original draft preparation: ZY, YL, and CJ. Study selection and data extraction: MX and ZC. Quality assessment: XZ and CJ. All authors have read and agreed to the published version of the manuscript.

## References

[1] Alzheimer's disease facts and figures. Alzheimers Dement. (2022) 18:700–89. 10.1002/alz.12638 35289055

[2] World Health Organization. Global Status Report on the Public Health Response to Dementia: Executive Summary. Geneva: World Health Organization (2021).

[3] Hugo J Ganguli M. Dementia and cognitive impairment: epidemiology, diagnosis, and treatment. Clin Geriatr Med. (2014) 30:421–42. 10.1016/j.cger.2014.04.001 25037289 PMC4104432

[4] Sachs-Ericsson N Blazer DG. The new DSM-5 diagnosis of mild neurocognitive disorder and its relation to research in mild cognitive impairment. Aging Ment Health. (2015) 19:2–12. 10.1080/13607863.2014.920303 24914889

[5] Jia L Du Y Chu L Zhang Z Li F Lyu D . Prevalence, risk factors, and management of dementia and mild cognitive impairment in adults aged 60 years or older in China: a cross-sectional study. Lancet Public Health. (2020) 5:e661–71. 10.1016/S2468-2667(20)30185-7 33271079

[6] Gauthier S Reisberg B Zaudig M Petersen RC Ritchie K Broich K . Mild cognitive impairment. Lancet. (2006) 367:1262–70. 10.1016/S0140-6736(06)68542-5 16631882

[7] Ward A Arrighi HM Michels S Cedarbaum JM. Mild cognitive impairment: disparity of incidence and prevalence estimates. Alzheimers Dement. (2012) 8:14–21. 10.1016/j.jalz.2011.01.002 22265588

[8] Langa KM Levine DA. The diagnosis and management of mild cognitive impairment: a clinical review. JAMA. (2014) 312:2551–61. 10.1001/jama.2014.13806 25514304 PMC4269302

[9] Eshkoor SA Hamid TA Mun CY Ng CK. Mild cognitive impairment and its management in older people. Clin Interv Aging. (2015) 10:687–93. 10.2147/CIA.S73922 25914527 PMC4401355

[10] Jongsiriyanyong S Limpawattana P. Mild cognitive impairment in clinical practice: a review article. Am J Alzheimers Dis Other Demen. (2018) 33:500–7. 10.1177/1533317518791401 30068225 PMC10852498

[11] Fink HA Jutkowitz E McCarten JR Hemmy LS Butler M Davila H . Pharmacologic interventions to prevent cognitive decline, mild cognitive impairment, and clinical Alzheimer-type dementia: a systematic review. Ann Intern Med. (2018) 168:39–51. 10.7326/M17-1529 29255847

[12] Cooper C Li R Lyketsos C Livingston G. Treatment for mild cognitive impairment: systematic review. Br J Psychiatry. (2013) 203:255–64. 10.1192/bjp.bp.113.127811 24085737 PMC3943830

[13] Deng M Wang XF. Acupuncture for amnestic mild cognitive impairment: a meta-analysis of randomised controlled trials. Acupunct Med. (2016) 34:342–8. 10.1136/acupmed-2015-010989 27491382

[14] Ho YS Zhao FY Yeung WF Wong GT Zhang HQ Chang RC. Application of acupuncture to attenuate immune responses and oxidative stress in postoperative cognitive dysfunction: what do we know so far? Oxid Med Cell Longev. (2020) 2020:9641904. 10.1155/2020/9641904 32148660 PMC7044481

[15] Huang L Yin X Li W Cao Y Chen Y Lao L . Effects of acupuncture on vascular cognitive impairment with no dementia: a randomized controlled trial. J Alzheimers Dis. (2021) 81:1391–401. 10.3233/JAD-201353 33935074 PMC8293636

[16] Kim H Kim HK Kim SY Kim YI Yoo HR Jung IC. Cognitive improvement effects of electro-acupuncture for the treatment of MCI compared with Western medications: a systematic review and Meta-analysis. BMC Complement Altern Med. (2019) 19:13. 10.1186/s12906-018-2407-2 30621676 PMC6325879

[17] Li W Wang Q Du S Pu Y Xu G. Acupuncture for mild cognitive impairment in elderly people: Systematic review and meta-analyses. Medicine (Baltimore). (2020) 99:e22365. 10.1097/MD.0000000000022365 32991455 PMC7523831

[18] He W Li M Han X Zhang W. Acupuncture for mild cognitive impairment and dementia: an overview of systematic reviews. Front Aging Neurosci. (2021) 13:647629. 10.3389/fnagi.2021.647629 34054504 PMC8160113

[19] Yu CC Wang Y Shen F Kong LH Wang YW Zhou H . High-frequency (50 Hz) electroacupuncture ameliorates cognitive impairment in rats with amyloid beta 1-42-induced Alzheimer's disease. Neural Regen Res. (2018) 13:1833–41. 10.4103/1673-5374.238620 30136700 PMC6128060

[20] Li W Kong LH Wang H Shen F Wang YW Zhou H . High-frequency electroacupuncture evidently reinforces hippocampal synaptic transmission in Alzheimer's disease rats. Neural Regen Res. (2016) 11:801–6. 10.4103/1673-5374.182708 27335565 PMC4904472

[21] Cai M Lee JH Yang EJ. Electroacupuncture attenuates cognition impairment via anti-neuroinflammation in an Alzheimer's disease animal model. J Neuroinflammation. (2019) 16:264. 10.1186/s12974-019-1665-3 31836020 PMC6909515

[22] Wetterslev J Jakobsen JC Gluud C. Trial sequential analysis in systematic reviews with meta-analysis. Bmc Med Res Methodol. (2017) 17:39. 10.1186/s12874-017-0315-7 28264661 PMC5397700

[23] Page MJ Moher D Bossuyt PM Boutron I Hoffmann TC Mulrow CD . PRISMA 2020 explanation and elaboration: updated guidance and exemplars for reporting systematic reviews. BMJ. (2021) 372:n160. 10.1136/bmj.n160 33781993 PMC8005925

[24] Shea BJ Reeves BC Wells G Thuku M Hamel C Moran J . AMSTAR 2: a critical appraisal tool for systematic reviews that include randomised or non-randomised studies of healthcare interventions, or both. BMJ. (2017) 358:j4008. 10.1136/bmj.j4008 28935701 PMC5833365

[25] Petersen RC Smith GE Waring SC Ivnik RJ Tangalos EG Kokmen E. Mild cognitive impairment: clinical characterization and outcome. Arch Neurol. (1999) 56:303–8. 10.1001/archneur.56.3.303 10190820

[26] Bondi MW Edmonds EC Jak AJ Clark LR Delano-Wood L McDonald CR . Neuropsychological criteria for mild cognitive impairment improves diagnostic precision, biomarker associations, and progression rates. J Alzheimers Dis. (2014) 42:275–89. 10.3233/JAD-140276 24844687 PMC4133291

[27] Guo LH Alexopoulos P Eisele T Wagenpfeil S Kurz A Perneczky R. The National Institute on Aging-Alzheimer's Association research criteria for mild cognitive impairment due to Alzheimer's disease: predicting the outcome. Eur Arch Psychiatry Clin Neurosci. (2013) 263:325–33. 10.1007/s00406-012-0349-0 22932720

[28] Sterne J Savović J Page MJ Elbers RG Blencowe NS Boutron I . RoB 2: a revised tool for assessing risk of bias in randomised trials. BMJ. (2019) 366:l4898. 10.1136/bmj.l4898 31462531

[29] Zhang YJ Cao HJ Li XL Yang XY Lai BY Yang GY . Cupping therapy versus acupuncture for pain-related conditions: a systematic review of randomized controlled trials and trial sequential analysis. Chin Med. (2017) 12:21. 10.1186/s13020-017-0142-0 28770000 PMC5525375

[30] Guyatt GH Oxman AD Schünemann HJ Tugwell P Knottnerus A GRADE. guidelines: a new series of articles in the Journal of Clinical Epidemiology. J Clin Epidemiol. (2011) 64:380–2. 10.1016/j.jclinepi.2010.09.011 21185693

[31] Yin ZH Wang LJ Cheng Y Chen J Hong XJ Zhao L . Acupuncture for chronic fatigue syndrome: an overview of systematic reviews. Chin J Integr Med. (2021) 27:940–6. 10.1007/s11655-020-3195-3 32279152

[32] Zheng M Chen K Huang J Mao CL. Clinical observation on the therapeutic effect of Fu Xiu Qi Zhi acupuncture for cognitive and Olfactony function. Asia-Pac Trad Med. (2021) 17:76–9. 10.11954/ytctyy.202110021

[33] Xu J Peng C. The clinical study of the electroacupuncture for treatment of amnestic mild cognitive impairment. Chin J General Pract. (2017) 15:393–6. 10.16766/j.cnki.issn.1674-4152.2017.03.009

[34] Tan TT Wang D Huang JK Zhou XM Yuan X Liang JP . Modulatory effects of acupuncture on brain networks in mild cognitive impairment patients. Neural Regen Res. (2017) 12:250–8. 10.4103/1673-5374.200808 28400807 PMC5361509

[35] Liu J. Electro-acupuncture intervention in essence deficiency mild cognitive impairment study of 1H-MR. World J Trad Chin Med. (2016):1093–5. 10.3969/j.issn.1673-7202.2016.05.03

[36] Du L. Study on Tongdu Tiaoshen Acupuncture on Mild Cognitive Impairment Patients with Serum BDNF Efficient. Anhui University of Chinese Medicine (2015). Available online at: https://kns.cnki.net/KCMS/detail/detail.aspx?dbname=CMFD201601&filename=1015365354.nh

[37] Zhu C Cai CS Xu B He CG Yang C Liang MJ. Clinical effect of Tongdu Tiaoshen acupuncture in treatment of amnestic Mild cognitive impairment. J Anhui Univ Trad Chin Med. (2015) 34:55–8. 10.3969/j.issn.2095-7246.2015.03.020

[38] Zhu C Cai CS Xu B. Clinical observation on mild cognitive impairment treated by Wenyang Bushen acupuncture. Clin J Trad Chin Med. (2014):795–7. 10.16448/j.cjtcm.2014.08.012

[39] Zhao L Zhang F Zhang H Zhao Y Zhou B Chen W Zhu M. Mild cognitive impairment disease treated with electroacupuncture: a multi-center randomized controlled trial. Zhongguo Zhen Jiu. (2012) 32:779–84. 10.13703/j.0255-2930.2012.09.004 23227678

[40] Liu X. Electro-acupuncture intervention in essence deficiency mild cognitive impairment study of 1H-MRS. Master, Xinjiang Medical University. (2010). Available online at: https://kns.cnki.net/KCMS/detail/detail.aspx?dbname=CMFD2011&filename=2010267082.nh

[41] Jin X. Electro-Acupuncture Intervention in Essence Deficiency Mild Cognitive Impairment Study of Clinical, Xinjiang Medical University. (2010). Available online at: https://kns.cnki.net/KCMS/detail/detail.aspx?dbname=CMFD2011&filename=2010266943.nh

[42] Wang Q. Clinical Study of Jiannao Bushen Acupuncture for MCI, Heilongjiang University of Traditional Chinese Medicine. (2009). Available online at: https://kns.cnki.net/KCMS/detail/detail.aspx?dbname=CMFD2009&filename=2009175471.nh

[43] MacPherson H White A Cummings M Jobst K Rose K Niemtzow R. Standards for reporting interventions in controlled trials of acupuncture: The STRICTA recommendations. STandards for Reporting Interventions in Controlled Trails of Acupuncture. Acupunct Med. (2002) 20:22–5. 10.1136/aim.20.1.22 11926601

[44] Cao H Wang Y Chang D Zhou L Liu J. Acupuncture for vascular mild cognitive impairment: a systematic review of randomised controlled trials. Acupunct Med. (2013) 31:368–74. 10.1136/acupmed-2013-010363 24123487 PMC3888636

[45] Vickers AJ Vertosick EA Lewith G MacPherson H Foster NE Sherman KJ . Acupuncture for chronic pain: update of an individual patient data meta-analysis. J Pain. (2018) 19:455–74. 10.1016/j.jpain.2017.11.005 29198932 PMC5927830

[46] Zhao L Chen J Li Y Sun X Chang X Zheng H . The long-term effect of acupuncture for migraine prophylaxis: a randomized clinical trial. Jama Intern Med. (2017) 177:508–15. 10.1001/jamainternmed.2016.9378 28241154

[47] Liao CC Liao KR Lin CL Li JM. Long-term effect of acupuncture on the medical expenditure and risk of depression and anxiety in migraine patients: a retrospective cohort study. Front Neurol. (2020) 11:321. 10.3389/fneur.2020.00321 32390934 PMC7193015

[48] Ahlberg R Skårberg K Brus O Kjellin L. Auricular acupuncture for substance use: a randomized controlled trial of effects on anxiety, sleep, drug use and use of addiction treatment services. Subst Abuse Treat Prev Policy. (2016) 11:24. 10.1186/s13011-016-0068-z 27451854 PMC4959048

[49] Jeon SW Kim KS Nam HJ. Long-term effect of acupuncture for treatment of tinnitus: a randomized, patient- and assessor-blind, sham-acupuncture-controlled, pilot trial. J Altern Complement Med. (2012) 18:693–9. 10.1089/acm.2011.0378 22747248

[50] Bian Z Liu B Moher D Wu T Li Y Shang H . Consolidated standards of reporting trials (CONSORT) for traditional Chinese medicine: current situation and future development. Front Med. (2011) 5:171–7. 10.1007/s11684-011-0132-z 21695622

