# Supplementary material for: Acupuncture for mild cognitive impairment: A systematic review with meta-analysis and trial sequential analysis
Source: Front Neurol. 2023 Jan 6;13:1091125. doi: 10.3389/fneur.2022.1091125 (PMC9853885; doi:10.3389/fneur.2022.1091125)
Supplement: Supplementary file 1 [file Table_1.DOCX]

**Appendix 1. Search strategies of each database.**

**PubMed**

#1 "Cognitive Dysfunction"[Mesh]

#2 "Cognitive Dysfunction"[Title/Abstract] OR "Cognitive Impairment"[Title/Abstract] OR "Age-Related Memory Disorders"[Title/Abstract] OR "Mild Cognitive Impairment"[Title/Abstract] OR "MCI"[Title/Abstract] OR "Mild Neurocognitive Disorder"[Title/Abstract] OR"Cognitive Decline"[Title/Abstract] OR "Mental Deterioration"[Title/Abstract]

#3 #1 OR #2

#4 "Acupuncture therapy"[Mesh]

#5 "acupuncture therapy"[Title/Abstract] OR "acupuncture"[Title/Abstract] OR "acupuncture-moxibustion"[Title/Abstract] OR "meridian*"[Title/Abstract] OR "acupoint*"[Title/Abstract] OR "warm needling"[Title/Abstract] OR "warm acupuncture"[Title/Abstract] OR "acupuncture plus moxibustion"[Title/Abstract] OR "electronic acupuncture"[Title/Abstract] OR "electro-acupuncture"[Title/Abstract] OR "electroacupuncture"[Title/Abstract] OR "fire acupuncture"[Title/Abstract] OR "auricular needle"[Title/Abstract] OR "scalp needle"[Title/Abstract] OR "abdominal needle"[Title/Abstract] OR "wrist ankle needle"[Title/Abstract] OR "triple puncture"[Title/Abstract] OR "dry needle"[Title/Abstract] OR "needle"[Title/Abstract] OR "body acupuncture"[Title/Abstract] OR "manual-acupuncture"[Title/Abstract]

#6 #4 OR #5

#7 "clinical trials, randomized"[Mesh Terms] OR "controlled clinical trials, randomized"[Mesh Terms] OR "clinical trials as topic"[MeSH Terms] OR "random allocation"[MeSH Terms] OR "therapeutic use"[MeSH Subheading]

#8 ("clinical"[Title/Abstract] AND "trial"[Title/Abstract]) OR "clinical trial"[Publication Type] OR "random*"[Title/Abstract]

#9 #7 OR #8

#10 #3 AND #6 AND #9

**EMBASE**

#1 'cognitive dysfunction'/exp/mj

#2 'cognitive dysfunction':ti,ab,kw OR 'cognitive impairment':ti,ab,kw OR 'age-related memory disorders':ti,ab,kw OR 'mild cognitive impairment':ti,ab,kw OR mci:ti,ab,kw OR 'mild neurocognitive disorder':ti,ab,kw OR 'cognitive decline':ti,ab,kw OR 'mental deterioration':ti,ab,kw

#3 #1 OR #2

#4 'acupuncture therapy'/exp/mj

#5 'acupuncture therapy':ti,ab,kw OR acupuncture:ti,ab,kw OR 'acupuncture moxibustion':ti,ab,kw OR meridian*:ti,ab,kw OR acupoint*:ti,ab,kw OR 'warm needling':ti,ab,kw OR 'warm acupuncture':ti,ab,kw OR 'acupuncture plus moxibustion':ti,ab,kw OR 'electronic acupuncture':ti,ab,kw OR 'electro acupuncture':ti,ab,kw OR electroacupuncture:ti,ab,kw OR 'fire acupuncture':ti,ab,kw OR 'auricular needle':ti,ab,kw OR 'scalp needle':ti,ab,kw OR 'abdominal needle':ti,ab,kw OR 'wrist ankle needle':ti,ab,kw OR 'triple puncture':ti,ab,kw OR 'dry needle':ti,ab,kw OR needle:ti,ab,kw OR 'body acupuncture':ti,ab,kw OR 'manual acupuncture':ti,ab,kw

#6 #4 OR #5

#7 'clinical trials, randomized'/exp/mj OR 'controlled clinical trials, randomized'/exp/mj OR 'clinical trials as topic'/exp/mj OR 'random allocation'/exp/mj

#8 clinical:ti,ab,kw OR trial:ti,ab,kw OR random*:ti,ab,kw

#9 #7 OR #8

#10 #3 AND #6 AND #9

**Web of science**

TS=(‘cognitive dysfunction’ OR ‘cognitive impairment’ OR ‘age-related memory disorders’ OR ‘mild cognitive impairment’ OR ‘mci’ OR ‘mild neurocognitive disorder’ OR ‘cognitive decline’ OR ‘mental deterioration’) AND TS=(acupuncture therapy’ OR ‘acupuncture’ OR ‘meridian’ OR ‘acupoint’ OR ‘warm needling’ OR ‘warm acupuncture’ OR ‘acupuncture plus moxibustion’ OR ‘moxibustion’ OR ‘electronic acupuncture’ OR ‘electro acupuncture’ OR ‘electroacupuncture:ti,ab,kw’ OR ‘'fire acupuncture’ OR ‘auricular needle’ OR ‘scalp needle’ OR ‘abdominal needle’ OR ‘wrist ankle needle’ OR ‘triple puncture’ OR ‘dry needle’ OR ‘needle’ OR ‘body acupuncture’ OR ‘manual acupuncture’ OR ‘moxibustion’) AND TS=(‘random*’ OR ‘clinical’ OR ‘trial’ )

**Cochrane Library**

#1 Mesh descriptor: [Cognitive Dysfunction]explode all trees

#2 cognitive dysfunction:ti,ab,kw OR cognitive impairment:ti,ab,kw OR age-related memory disorders:ti,ab,kw OR mild cognitive impairment:ti,ab,kw OR mci:ti,ab,kw OR mild neurocognitive disorder:ti,ab,kw OR cognitive decline:ti,ab,kw OR mental deterioration:ti,ab,kw

#3 #1 OR #2

#4 Mesh descriptor: [acupuncture therapy] explode all trees;

#5 acupuncture therapy:ti,ab,kw OR acupuncture:ti,ab,kw OR acupuncture moxibustion:ti,ab,kw OR meridian*:ti,ab,kw OR acupoint*:ti,ab,kw OR warm needling:ti,ab,kw OR warm acupuncture:ti,ab,kw OR acupuncture plus moxibustion:ti,ab,kw OR electronic acupuncture:ti,ab,kw OR electro acupuncture:ti,ab,kw OR electroacupuncture:ti,ab,kw OR fire acupuncture:ti,ab,kw OR auricular needle:ti,ab,kw OR scalp needle:ti,ab,kw OR abdominal needle:ti,ab,kw OR wrist ankle needle:ti,ab,kw OR triple puncture:ti,ab,kw OR dry needle:ti,ab,kw OR needle:ti,ab,kw OR body acupuncture:ti,ab,kw OR manual acupuncture:ti,ab,kw

#6 #4 OR #5

#7 Mesh descriptor: [clinical trials, randomized]

#8Mesh descriptor: [controlled clinical trials, randomized]

#9Mesh descriptor: [clinical trials as topic]

#10Mesh descriptor: [random allocation] explode all trees;

#11 clinical:ti,ab,kw OR trial:ti,ab,kw OR random*:ti,ab,kw

#12 #7 OR #8 OR #9 OR #10 OR #11

#13 #3 AND #6 AND #12

**CNKI**

(TKA=(‘针刺’+‘针灸’+‘体针’+‘手针’+‘电针’+‘温针’+‘头皮针’+‘头针’+‘耳针’+‘穴位’+‘火针’+‘腹针’+‘浮针’+‘三棱针’+‘九针’+‘经络’+‘经皮电刺激’+‘眼针’+‘舌针’+‘腕踝针’+‘刃针’+‘针刀’+‘刺络’+‘干针’) OR SU=(‘针刺疗法' + '针刺' + '针灸疗法’)) AND (TKA=(‘轻度认知障碍’+‘轻度认知功能障碍’+‘轻度认知损害’+‘轻度认知损伤’+‘轻度神经认知障碍’+‘认知减退’+‘精神衰退’)) AND (TKA=(‘随机’+‘对照’) OR SU=(‘随机对照试验’))

**WF**

(题名或关键词:(轻度认知障碍 OR 轻度认知功能障碍 OR 轻度认知损害 OR 轻度认知损伤 OR 轻度神经认知障碍)) and (题名或关键词:(针刺 OR 针灸 OR 体针 OR 手针 OR 电针 OR 温针 OR 头皮针 OR 头针 OR 耳针 OR 穴位 OR 火针 OR 腹针 OR 浮针 OR 三棱针 OR 九针 OR 经络 OR 经皮电刺激 OR 眼针 OR 舌针 OR 腕踝针 OR 刃针 OR 针刀 OR 刺络 OR 干针) OR 主题:(针刺疗法 OR 针灸疗法 OR 针刺)) and (题名或关键词:(随机 OR 对照) OR 主题:(随机对照试验))

**Chongqing VIP**

M=(轻度认知障碍 OR 轻度认知功能障碍 OR 轻度认知损害 OR 轻度认知损伤 OR 轻度神经认知障碍 OR 认知减退 OR 精神衰退) and M=(针刺 OR 针灸 OR 体针 OR 手针 OR 电针 OR 温针 OR 头皮针 OR 头针 OR 耳针 OR 穴位 OR 火针 OR 腹针 OR 浮针 OR 三棱针 OR 九针 OR 经络 OR 经皮电刺激 OR 眼针 OR 舌针 OR 腕踝针 OR 刃针 OR 针刀 OR 刺络 OR 干针) and R=(随机 OR 对照)

**CBM**

1 "认知障碍" [加权:扩展]

2 "轻度认知障碍"[常用字段:智能] OR "轻度认知功能障碍"[常用字段:智能] OR "轻度认知损害"[常用字段:智能] OR "轻度认知损伤"[常用字段:智能] OR "轻度神经认知障碍"[常用字段:智能] OR "认知减退"[常用字段:智能] OR "精神衰退"[常用字段:智能]

3 1 OR 2

4 "针刺疗法"[加权:扩展] OR "针刺"[加权:扩展] OR "针灸疗法"[加权:扩展]

5 "针刺"[常用字段:智能] OR "针灸"[常用字段:智能] OR "体针"[常用字段:智能] OR "手针"[常用字段:智能] OR "电针"[常用字段:智能] OR "温针"[常用字段:智能] OR "头皮针"[常用字段:智能] OR "头针"[常用字段:智能] OR "耳针"[常用字段:智能] OR "穴位"[常用字段:智能] OR "火针"[常用字段:智能] OR "腹针"[常用字段:智能] OR "浮针"[常用字段:智能] OR "三棱针"[常用字段:智能] OR "九针"[常用字段:智能] OR "经络"[常用字段:智能] OR "经皮穴位电刺激"[常用字段:智能] OR "眼针"[常用字段:智能] OR "舌针"[常用字段:智能] OR "腕踝针"[常用字段:智能] OR "刃针"[常用字段:智能] OR "针刀"[常用字段:智能] OR "刺络"[常用字段:智能] OR "干针"[常用字段:智能] OR "灸"[常用字段:智能]

6 4 OR 5

7 "随机对照试验"[不加权:扩展]

8 "随机"[常用字段:智能] OR "对照"[常用字段:智能]

9 7 OR 8

10 3 AND 6 AND 9

**Appendix 2. Full-text articles excluded with reasons**

| Full-text articles excluded | Reasons |
| --- | --- |
| Mai, W. 2021 [1] | Ineligible intervention |
| Wang, H. 2021 [2] | Ineligible intervention |
| Wang, Y. 2021 [3] | Ineligible intervention |
| Wang, H. 2020 [4] | Ineligible intervention |
| Li, S. 2020 [5] | Ineligible intervention |
| Zhao, L. 2019 [6] | Ineligible intervention |
| Liu, C. 2017 [7] | Ineligible intervention |
| Yang, X. 2016 [8] | Ineligible intervention |
| Zhu, C. 2015 [9] | Ineligible intervention |
| Li, H. 2020 [10] | Ineligible intervention |
| Kim, J.H. 2021 [11] | Ineligible outcome |
| Shan, Y. 2018 [12] | Ineligible outcome |
| Jia, B. 2015 [13] | Ineligible outcome |
| Xu, M.Z. 2013 [14] | Ineligible outcome |
| Wang, Z. 2012 [15] | Ineligible outcome |
| Jiang, C.G. 2012 [16] | Ineligible outcome |
| Cui, S.Y. 2011 [17] | Ineligible outcome |
| Liu, X.P. 2010 [18] | Ineligible outcome |
| Liu, J. 2009a [19] | Ineligible outcome |
| Liu, J. 2009b [20] | Duplicate content |

**References**

1. Mai, W.; Zhang, A.; Liu, Q.; Tang, L.; Wei, Y.; Su, J.; Duan, G.; Teng, J.; Nong, X.; Yu, B.; et al. Effects of moxa cone moxibustion therapy on cognitive function and brain metabolic changes in MCI patients: A pilot (1)H-MRS study**.** *Front. Aging Neurosci.* **2022**, *14*, 773687**.**

2. Wang, H.; Li, S.; Hu, Q.; Yu, H.; Zhang, H. Effect on moxibustion on memory function and related serum protein markers in patients with amnestic mild cognitive impairment. *Acupuncture Research*. **2021**, *46*, 794-9

3. Wang, Y.; Liu, J.; Yang, T.; Liu, J.; Qin, H.; Li, J. Clinical Study on Acupoint Catgut Embedding Therapy for Abdominal Obesity Complicated with Mild Cognitive Impairment. *J Guangzhou Univer Trad Chin Med* **2021**, *38*, 311-316**.**

4. Wang, H.; Hu, Q.; Yu, H.; Wang, L.; Zhang, H. Clinical effect and safety of moxibustion therapy in treatment of mild cognitive impairment: a multi-center randomized controlled trials. *Acupuncture Research*. **2020,** *45*, 794-799.

5. Li, S. Effects of moxibustion combined with cognitive training on cognitive function and serum levels of Aβ 1-42, Tau, P-tau in patients with Mild Cognitive Impairment, Chengdu University of Traditional Chinese Medicine, 2020.

6. Zhao, L.; Yu B.; Nong, X.; Wen, J.; Liu, Q; Tang, L; Liang, J.; Zhou, L.; Mai, W.; Li, Q.; et al. Correlation between the changes of 8-isoprostaglandin F2α in serum and urine and cognitive function in patients with mild cognitive impairment and the effect of moxibustion. *Chin J Gerontol* **2019**, *39*, 3699-3703**.**

7. Liu, C. Clinical evaluation of “San Cai of Health of Brain” moxibustion for treatment of Mild Cognitive Impairment, 2017.

8. Yang, X. Clinical evaluation on the therapeutic effects of the moxibustion of San Cai of Health of Brain for the treatment of mild cognitive impairment, Chengdu University of Traditional Chinese Medicine, 2016.

9. Zhu, C. Summary of CAI Shengchao's academic thoughts and clinical experiences and clinical research on the moxibustion treatment named "Wenyangbushen" for the treatment of mild cognitive impairment, Nanjing University of Chinese Medicine, 2015.

10. Li, H.; Wang, Z.; Yu, H.; Pang, R.; Ni, H.; Li, C.; Li, K.; Canevelli, M. The Long-Term effects of acupuncture on hippocampal functional connectivity in aMCI with hippocampal atrophy: A randomized longitudinal fMRI study. 2020, 2020, 6389368.

11. Kim, J.H.; Cho, M.R.; Shin, J.C.; Park, G.C.; Lee, J.S. Factors contributing to cognitive improvement effects of acupuncture in patients with mild cognitive impairment: A pilot randomized controlled trial**.** *Trials* **2021**, *22*, 341**.**

12. Shan, Y.; Wang, J.J.; Wang, Z.Q.; Zhao, Z.L.; Zhang, M.; Xu, J.Y.; Han, Y.; Li, K.C.; Lu, J. Neuronal specificity of acupuncture in alzheimer's disease and mild cognitive impairment patients: A functional MRI study**.** *Evid Based Complement Alternat Med* **2018**, *2018*, 7619197**.**

13. Jia, B.; Liu, Z.; Min, B.; Wang, Z.; Zhou, A.; Li, Y.; Qiao, H.; Jia, J. The Effects of Acupuncture at Real or Sham Acupoints on the Intrinsic Brain Activity in Mild Cognitive Impairment Patients**.** *Evid Based Complement Alternat Med* **2015**, *2015*, 529675**.**

14. Xu, M.Z. To explore the bidirectional adjustment mechanism of acupuncture at Taixi point based on fMRI, Guangzhou University of Chinese Medicine, 2013.

15. Wang, Z.; Nie, B.; Li, D.; Zhao, Z.; Han, Y.; Song, H.; Xu, J.; Shan, B.; Lu, J.; Li, K. Effect of acupuncture in mild cognitive impairment and Alzheimer disease: A functional MRI study**.** *PLoS One* **2012**, *7*, e42730**.**

16. Jiang, C.G.; Cui, S.Y.; Nie, B.B.; Tang, C.Z.; Zhang, J.W. Effect of needling taixi (KI3) acupoint and sham point on functional magnetic resonance imaging in mild cognitive impairment patients**.** *Journal of New Chinese Medicine* **2012**, *44*, 93-95**.**

17. Cui, S.Y. Effect of Acupuncture at KI3 on Cerebral Function Imaging, Guangzhou University of Chinese Medicine, 2011.

18. Liu, X.P. Electro-acupuncture intervention in essence deficiency mild cognitive impairment study of 1H-MRS, Xinjiang Medical University, 2010.

19. Liu, J. Electro-acupuncture intervention in essence deficiency mild cognitive impairment study of 1H-MRS, Xinjiang Medical University, 2009.

20. Liu, J.; Liu Z.Y. Clinical Observations on Electroacupuncture Treatment for Mild Cognitive Impairment of Kidney Essence Deficiency Type. Shanghai J Acu-Mox 2009, 28, 319-321.
